# The Association Between Composite Healthy Lifestyle Score and Type 2 Diabetes Risk in the Korean Population: The Korean Genome and Epidemiology Study

**DOI:** 10.3390/nu18020273

**Published:** 2026-01-14

**Authors:** Daeyun Kim, Minji Kang, Dongmin Kim, Juyoung Park, Jihye Kim

**Affiliations:** 1 Department of Genetics and Biotechnology, College of Life Sciences, Kyung Hee University, Yongin 17104, Republic of Korea; daeyun5405@khu.ac.kr (D.K.); kdmpre@khu.ac.kr (D.K.); 2 Department of Food and Nutrition, Duksung Women’s University, Seoul 01369, Republic of Korea; mjkang@duksung.ac.kr; 3 Department of Statistics, College of Natural Sciences, Yeungnam University, Gyeongsan 38541, Republic of Korea; jystat@yu.ac.kr

**Keywords:** healthy lifestyle score, T2D, cohort study

## Abstract

**Background/Objectives**: Modifiable lifestyle factors, particularly diet, are important for preventing type 2 diabetes (T2D); however, the evidence regarding this from prospective studies is limited in the Asian population. We therefore evaluated whether a diet-inclusive healthy lifestyle score (HLS) predicts incident T2D in a community-based cohort. **Methods**: We analyzed 7185 T2D-free adults from the KoGES Ansan–Ansung cohort, constructing the HLS (range: 0–5) based on five lifestyle factors: non-smoking, ≥30 min/day of moderate-to-vigorous physical activity, low-risk alcohol consumption (≤40 g/day for men; ≤20 g/day for women), BMI of 18.5–24.9 kg/m^2^, and a healthy diet, defined as a healthy plant-based diet index within the top 40th percentile. Cox proportional hazards regression models were employed to examine the association between HLS and incident T2D risk. **Results**: During a median follow-up of 17.5 years, 1223 cases of T2D were identified. Compared to individuals with a score of 0 or 1, those with a score of 5 had a 56% lower risk of T2D after adjustment for potential confounders (HR: 0.44, 95% CI: 0.32–0.62), and these associations remained consistent across subgroups stratified by age, sex, family history of T2D, hypertension, and residential area. However, the association was stronger among non-users of anti-diabetic medication than among users. **Conclusions**: Adherence to a healthier lifestyle, as indicated by a higher HLS, was significantly associated with a reduced risk of developing T2D among Korean adults. These findings underscore the importance of promoting integrated healthy lifestyle behaviors to prevent T2D.

## 1. Introduction

Type 2 diabetes (T2D) is a chronic metabolic disorder characterized by impaired insulin secretion or action, resulting in sustained hyperglycemia [1]. In 2024, an estimated 589 million adults aged 20–79 years were living with diabetes, and this total is projected to reach 853 million by 2050 [2]. Adults with T2D have approximately double the cardiovascular disease risk, and high blood glucose is estimated to account for 11% (10–12%) of cardiovascular-related deaths globally [3].

The results of epidemiologic studies have demonstrated that a substantial proportion of T2D is potentially preventable through the combined modification of lifestyle behaviors [4,5,6]. A meta-analysis identified robust associations between incident T2D and multiple modifiable exposures, including adiposity, smoking, physical activity, alcohol intake, and diet-related factors [7]. Given the clustering of these behaviors, composite lifestyle scores have been used to capture their joint impact. In the Nurses’ Health Study, adherence to a low-risk profile (healthy body weight, higher-quality diet, regular physical activity, non-smoking, and moderate alcohol intake) was associated with a 91% lower T2D risk [8]. More recently, a dose–response meta-analysis of 30 cohort comparisons reported an 80% lower incident T2D risk for those with the highest versus lowest adherence to multiple low-risk behaviors [4]. Nevertheless, the evidence on multi-behavior lifestyle profiles remains dominated by predominantly Western cohorts, and large amounts of prospective data from Asian populations are comparatively limited [9].

Furthermore, most prior studies in Asian populations have omitted diet [9,10]—an essential component of T2D risk—because of limited dietary assessment, or have evaluated diet using only a few food items rather than overall diet quality [9,10,11,12,13,14,15,16,17,18]. Diet has been approximated using a small set of frequency-based items (e.g., daily vegetables, fruits, and wheat, with less than daily red meat intake) [9] or broad food group categories (e.g., higher vegetables/fruits/whole grains and lower meat intake) without a validated diet-quality index [10]. Incorporating validated indices that more comprehensively assess diet may strengthen the construct validity of lifestyle scores. For example, the healthful plant-based diet index (hPDI) evaluates overall diet quality by positively scoring healthy plant foods and inversely scoring less healthy plant and animal foods and has been associated with a lower risk of incident T2D in large prospective cohorts, including Korean cohorts [19,20,21].

Koreans are more vulnerable to T2D than Western populations, despite a lower incidence of obesity, which is a major T2D risk factor, due to differences in lifestyle patterns, including eating habits, metabolic responses to diet, and genetic variants [22,23,24]. For instance, previous studies reported ethnic differences in T2D risk associated with a priori-defined dietary pattern scores [24]; therefore, it is important to investigate whether associations observed in Western populations are consistent with responses in Korean populations.

In this context, we aimed in this study to evaluate the combined effects of key lifestyle factors—including smoking, physical activity, alcohol consumption, BMI, and dietary habits—on the risk of developing T2D by constructing a healthy lifestyle score (HLS) using data from a population-based prospective cohort study. We hypothesized that higher adherence to the HLS would be associated with a lower incident T2D risk among Korean adults.

## 2. Materials and Methods

### 2.1. Study Population

The data were obtained from the Korean Genome and Epidemiology Study (KoGES), a population-based cohort study which investigates non-communicable chronic diseases such as T2D and hypertension [25]. A total of 10,030 individuals (40–79 years of age) were recruited from the Ansan and Ansung areas between 2001 and 2002 and were followed up every two years, with the most recent follow-up occurring in 2019–2020 [25]. The Institutional Review Board of Kyung Hee University (KHGIRB-25-278(RA)) approved the study protocol, and participants provided written informed consent.

**Figure 1:**
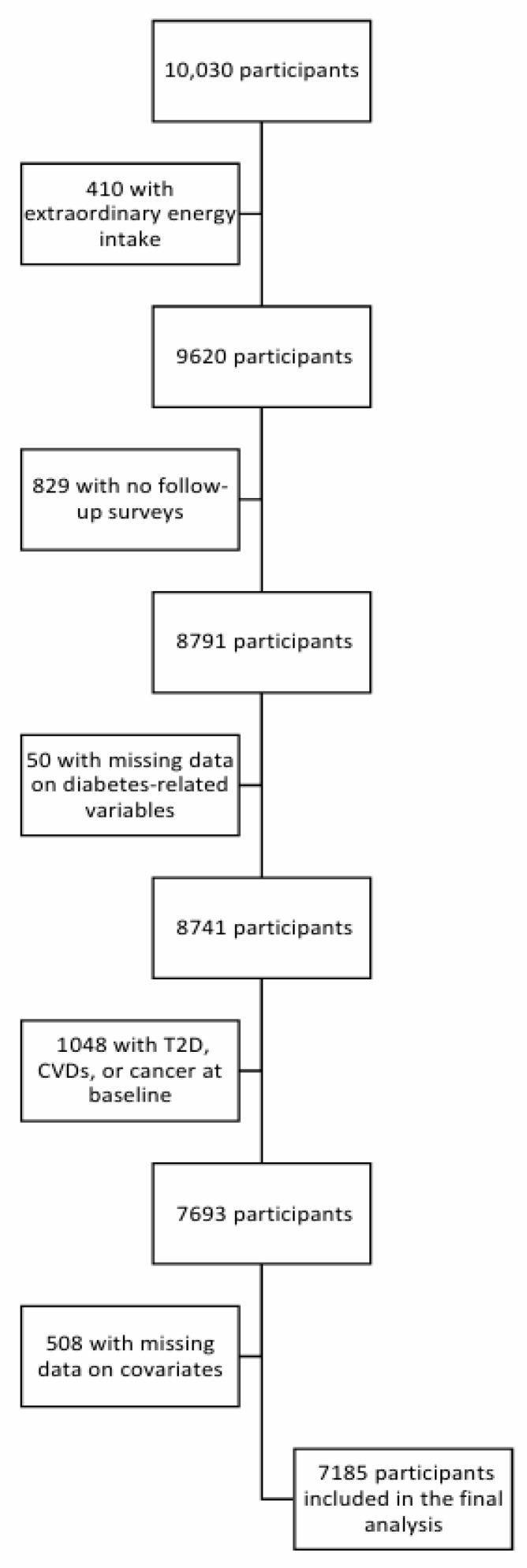
Flow chart of the study population selection.

Individuals with an energy intake outside the 500–5000 kcal range (n = 410); those who did not participate in follow-up surveys (n = 829); those with missing diabetes-related variable data (n = 50); those with T2D, cardiovascular diseases (myocardial infarction, congestive heart failure, coronary artery disease, and cerebrovascular disease) or cancers (n = 1048); and those with missing data on covariates (n = 508) were excluded. Finally, 7185 participants were included in this study (Figure 1).

### 2.2. Ascertaining a Healthy Lifestyle Score

Lifestyle factors were assessed using a standardized questionnaire administered at baseline, and anthropometric measurements were obtained at the study visit by trained staff [25]. A healthy lifestyle score (HLS) was constructed by dichotomizing five lifestyle factors (smoking, physical activity, alcohol consumption, BMI, and diet) and summing the resulting binary indicators (range: 0–5). For each component, participants received 1 point if they met the predefined low-risk criterion and 0 points otherwise. A higher total score indicated greater adherence to healthy lifestyle behaviors [26,27].

Smoking status was derived from the questionnaire items assessing lifetime and current cigarette use (e.g., “Have you ever smoked in your entire life?” and “Do you currently smoke cigarettes?”) [25], with participants who answered “No” to both classified as never being smokers and assigned 1 point; former and current smokers were assigned 0 points. Physical activity was assessed using the baseline questionnaire, with participants engaging in ≥30 min per day of moderate-to-vigorous physical activity assigned 1 point and those reporting <30 min per day assigned 0 points [25,27]. Alcohol intake was assessed using intake frequency and amount questions [25], with a healthy status assigned to participants who were never or former drinkers, or to current drinkers whose intake did not exceed 40 g/day for men or 20 g/day for women; participants exceeding these thresholds received 0 points [28]. BMI was calculated as weight (kg) divided by height squared (m^2^), with a value between 18.5 and 24.9 kg/m^2^ considered healthy (1 point); values outside this range were assigned 0 points [29]. Dietary intake was assessed using a validated food frequency questionnaire administered at baseline and during the second follow-up [25,30]. We calculated the healthful plant-based diet index (hPDI) or unhealthful plant-based diet index (uPDI) scores following the established scoring algorithm [19,21,31]; briefly, individual FFQ items were aggregated into 17 food groups based on nutrient and culinary similarities, which were then categorized into three larger groups: healthy plant foods, less-healthy plant foods, and animal foods. For each of the 17 food groups, participants were ranked into quintiles of intake and assigned scores from 1 (lowest) to 5 (highest). For hPDI, healthy plant food groups were positively scored (1–5), whereas less healthy plant and animal food groups were scored in reverse (5–1). For uPDI, healthy plant food groups were scored in reverse (5–1), whereas less-healthy plant and animal food groups were positively scored (1–5). Component scores were summed to create the total index score; higher scores indicate greater adherence to the corresponding plant-based dietary pattern. For the HLS dietary component, a healthy diet was defined as the highest 40% of the cohort hPDI distribution or the lowest 40% of the cohort uPDI distribution (in case of sensitivity analysis, replacing hPDI with uPDI), and participants meeting this criterion received 1 point; all others received 0 points [21].

### 2.3. Ascertaining T2D

The diagnostic criteria for new-onset T2D included the presence of one or more of the following [32]: a previous T2D diagnosis, a fasting glucose level ≥ 126 mg/dL, and the use of glucose-lowering medications or insulin treatment.

### 2.4. Statistical Analysis

Baseline characteristics were presented as n (%) for categorical variables and mean (SD) for continuous variables. Person-years of follow-up were calculated from the baseline survey date to the date of T2D diagnosis or the last follow-up. Cox proportional hazards regression models were used to estimate the association between the HLS and incident T2D risk, with results presented as hazard ratios (HRs) and 95% confidence intervals (CIs). Model 1 was adjusted for age and sex [21,25,33]. Additionally, model 2 was further adjusted for residential area, educational level, family T2D history, hypertension status, total energy intake, menopausal status, use of hormone replacement therapy, and fasting glucose level.

Stratified analyses were conducted according to age (<49/≥49 years), sex (men/women), family T2D history (yes/no), hypertension status (yes/no), BMI (<25/≥25 kg/m^2^), anti-diabetic medication (yes/no), and residential area (urban/rural) to assess potential effect modification. Several sensitivity analyses were also performed: First, the HLS was recalculated by substituting the hPDI with the uPDI. Second, individuals who developed T2D within the first 2 or 4 years of follow-up were excluded to minimize reverse causality. Leave-one-out analyses were also performed by sequentially excluding each of the five lifestyle factors to assess the relative contribution of individual components to T2D risk. The proportional hazards assumption test showed no violation. All statistical analyses were performed using SAS version 9.4 (SAS Institute, Cary, NC, USA) and a two-sided *p*-value < 0.05 was considered statistically significant.

## 3. Results

The study population’s baseline characteristics are shown in Table 1. Among the 7185 participants, those in the highest HLS category tended to be older, more likely to be women, less likely to reside in urban areas, less educated, and reported lower total energy intake. In addition, we compared the basic characteristics between overall-cohort and included participants (Supplementary Table S1). Participants from the overall cohort were older than the included participants, but we did not find a large difference that would raise concerns about whether the sample was representative of the entire cohort.

During a median 17.5 years of follow-up, a total of 1223 incident cases of T2D were identified. As shown in Table 2, in the multivariable adjusted model, participants with the highest HLS had a 56% lower risk of developing T2D (95% CI: 0.32–0.62) compared with those with the lowest, with a significant linear trend across increasing HLS (*p* for trend < 0.0001). For a 1-point increment in HLS, the HR for incident T2D was 0.85 (95% CI: 0.69–1.04).

In the stratified analyses according to age, sex, family T2D history, hypertension status, BMI, and residential area, the associations between HLS and T2D risk were generally consistent (Table 3); however, the inverse association was stronger in participants without anti-diabetic medication use (HR: 0.48, 95% CI, 0.26–0.90) than in those with anti-diabetic medication use (*p* for interaction = 0.011).

Sensitivity analyses showed consistent findings; when the hPDI was replaced with the uPDI, or when T2D cases identified during the first 2 or 4 years of follow-up were excluded, the results remained materially unchanged (Table 4).

Leave-one-out analyses were conducted to assess the relative contribution of each lifestyle factor to T2D risk by omitting one at a time. In all analyses, higher HLS was associated with a lower incident T2D risk (Table 5). The strongest inverse association was observed when diet was excluded (HR comparing the highest versus the lowest HLS category: 0.42; 95% CI: 0.32–0.53), with a significant inverse linear trend (*p* for trend <0.0001) while the weakest association was found when BMI was excluded (HR: 0.82; 95% CI: 0.65–1.04), with no evidence of a linear trend (*p* for trend = 0.2145).

## 4. Discussion

This study found an inverse association between HLS and incident T2D, with participants having healthier lifestyle profiles experiencing lower risk after multivariate adjustment compared to those with the least healthy profiles. These results were highly consistent across stratified subgroups by age, sex, family T2D history, hypertension status, and residential area, with little evidence of heterogeneity across these strata. By contrast, analyses stratified by anti-diabetic medication use yielded effect modification evidence (*p* for interaction < 0.05); notably, the association was stronger among non-users.

Consistent with our findings, a recent meta-analysis of prospective cohorts reports substantial reductions in incident T2D among individuals with the healthiest versus the least healthy lifestyle profiles, with the largest reduction observed when multiple behaviors are optimized [4]. Across Asia, Europe, and the Americas, composite lifestyle scores that integrate diet, physical activity, smoking, alcohol use, and related behaviors show a graded, inverse association with T2D incidence. For example, in the UK Biobank, a four-component HLS (non-smoking, moderate alcohol use, adequate physical activity, and higher diet quality) was inversely associated with diabetes risk, with lower risk among participants adhering to three or four healthy behaviors compared with those adhering to few or none [34]. Similarly, analyses from the Nurses’ Health Study and the Health Professionals Follow-Up Study found that adhering to a healthy lifestyle—never smoking, having an adequate BMI, regular moderate-to-vigorous physical activity, moderate alcohol use, and higher diet quality—was associated with longer life expectancy free of major chronic disease, including T2D [35].

Building on the comparative evidence above, the inverse association we observed between HLS and incident T2D is consistent with the biological effects of each lifestyle factor on glucose regulation and insulin resistance. In our study, overall diet quality was assessed with the hPDI, which prioritizes higher intake of whole grains, fruits, vegetables, legumes, and nuts while limiting less-healthy plant and animal foods. Such patterns lower postprandial glycemic load, support fiber- and micronutrient-mediated metabolic signaling, and reduce hepatic and visceral fat, thereby improving insulin sensitivity and supporting β-cell function. Physiologically, higher intake of fiber-rich, minimally processed plant foods may improve glucose homeostasis via gut microbiome and incretin-related signaling, while lower glycemic load and reduced ectopic fat accumulation may mitigate hepatic insulin resistance and β-cell metabolic burden [36,37]. These pathways may help to explain why the HLS–T2D association’s magnitude differed when the dietary component was omitted in leave-one-out analyses. Regular physical activity also supports insulin sensitivity in skeletal muscle and mitochondrial biogenesis while reducing visceral and intrahepatic fat [38,39].

On the other hand, accumulating evidence suggests that smoking contributes to an increased T2D risk by promoting pancreatic islet β-cell senescence and exacerbating insulin resistance [40,41]. Excessive alcohol consumption induces pancreatic β-cell dysfunction and impaired glucose homeostasis, thereby contributing to an increased T2D risk, particularly among adult men [42,43]. Additionally, BMI—primarily a proxy for adiposity and potential downstream diet and physical activity marker—is associated with elevated insulin resistance and low-grade inflammation, consistent with the robust prospective association of overweight/obesity with incident T2D [44,45].

Taken together, the five factors—diet quality, smoking, physical activity, alcohol use, and adiposity—exert partially overlapping yet non-identical influences on glucose regulation and insulin resistance. More broadly, these lifestyle factors likely converge on key processes in T2D pathophysiology, including hepatic and skeletal muscle insulin resistance, chronic low-grade inflammation, and progressive β-cell dysfunction, consistent with the graded inverse association across HLS categories [4,46]. Accordingly, we suggest that coordinated improvements in five lifestyle factors related to T2D are likely to yield cumulative—or at least additive—benefits for reducing T2D risk rather than targeting a single behavior, as reflected in the stepwise decline in risk across HLS categories.

The strengths of this study include using data from a community-based cohort, validated and repeated dietary assessments, and a relatively long follow-up period to evaluate the association between HLS and T2D incidence risk. Unlike previous studies, in this study we incorporated the hPDI into the assessment of a healthy lifestyle, as it is validated as a T2D risk factor and as an indicator of overall diet quality [21,47]. However, our study has several limitations: First, HLS was derived solely from baseline data, while dietary assessment was limited to baseline and second follow-up measurements, thereby not accounting for potential changes during the follow-up period. Second, self-reported instruments, such as FFQ, may introduce measurement errors. Third, there may still be residual confounders, despite adjusting important confounders.

## 5. Conclusions

Adherence to a healthier lifestyle was associated with lower T2D risk in a community-based cohort of Korean adults. These findings emphasize the necessity of integrated, multi-behavior prevention strategies, suggesting cumulative benefits when various lifestyle factors are improved together. Future research is warranted to test whether HLS-oriented interventions lower diabetes incidence and to reveal the biological pathway linking these modifiable lifestyle factors to T2D and related metabolic diseases.

## Figures and Tables

**Table 1:** Baseline characteristics of participants by healthy lifestyle score category *.

	Healthy Lifestyle Score				
	0~1	2	3	4	5
Participants	548 (7.6)	1615 (22.5)	2611 (36.3)	1960 (27.3)	451 (6.3)
Age, years	47.7 (7.1)	50.2 (8.4)	51.6 (8.6)	53.0 (9.0)	55.0 (8.8)
Sex					
Women	51 (9.3)	497 (30.8)	1453 (55.7)	1360 (69.4)	348 (77.2)
Residential area					
Rural (Ansung)	113 (20.6)	573 (35.5)	1265 (48.5)	1138 (58.1)	307 (68.1)
Urban (Ansan)	435 (79.4)	1042 (64.5)	1346 (51.6)	822 (41.9)	144 (31.9)
Educational level, years					
≤6	70 (12.8)	330 (20.4)	855 (32.8)	775 (39.5)	218 (48.3)
7–12	321 (58.6)	996 (61.7)	1422 (54.5)	985 (50.3)	202 (44.8)
>12	157 (28.7)	289 (17.9)	334 (12.8)	200 (10.2)	31 (6.9)
Smoking status					
Never	20 (3.7)	541 (33.5)	1618 (62.0)	1593 (81.3)	451 (100)
Former	186 (33.9)	390 (24.2)	390 (14.9)	152 (7.8)	0
Current	342 (62.4)	684 (42.4)	603 (23.1)	215 (11.0)	0
Physical activity					
0	358 (65.3)	818 (50.7)	848 (32.5)	222 (11.3)	0
<30 min/d	116 (21.2)	277 (17.2)	248 (9.5)	73 (3.7)	0
≥30 min/d	74 (13.5)	520 (32.2)	1515 (58.0)	1665 (85.0)	451 (100)
Alcohol consumption, g/d					
Men	45.0 (37.5)	28.0 (32.0)	18.9 (21.1)	14.7 (20.1)	11.2 (9.8)
Women	32.7 (30.7)	6.2 (9.8)	4.6 (7.9)	3.4 (4.7)	2.1 (2.3)
BMI, kg/m^2^	26.2 (3.0)	25.4 (3.2)	24.5 (3.2)	23.7 (2.8)	22.7 (1.6)
hPDI, score					
Men	43.6 (4.9)	45.5 (5.7)	47.9 (6.0)	52.1 (5.1)	54.6 (3.6)
Women	44.1 (4.4)	44.4 (4.7)	46.5 (5.7)	49.5 (6.5)	54.7 (3.3)
Family T2D history	64 (11.7)	165 (10.2)	296 (11.3)	206 (10.5)	36 (8.0)
Hypertension status	75 (13.7)	202 (12.5)	360 (13.8)	261 (13.3)	57 (12.6)
Total energy intake, kcal/d	2039 (529)	1975 (584)	1928 (596)	1904 (656)	1793 (583)
Menopause status	32 (62.8)	272 (54.7)	847 (58.3)	839 (61.7)	245 (70.4)
Hormone replacement therapy use	2 (3.9)	22 (4.4)	72 (5.0)	63 (4.6)	18 (5.2)
T2D incidence	138 (25.2)	295 (18.3)	461 (17.7)	276 (14.1)	53 (11.8)
Fasting glucose level, mg/dL	92.0 (10.5)	89.1 (9.4)	88.3 (9.7)	86.9 (8.8)	86.7 (8.6)

* Data are expressed as n (%) or mean (SD). Abbreviations: BMI, body mass index; hPDI, healthy plant-based diet index; T2D, type 2 diabetes.

**Table 2:** HR (95% CI) of incident T2D according to healthy lifestyle score category.

		Healthy Lifestyle Score						
	No. of Cases/Person-Years	0~1	2	3	4	5	*p* for Trend	Per 1-Point Increment
Model 1 *	1223/98,401	ref	0.65 (0.52–0.80)	0.58 (0.47–0.71)	0.42 (0.33–0.52)	0.31 (0.22–0.44)	<0.0001	0.77 (0.72–0.81)
Model 2 ^†^	1223/98,401	ref	0.85 (0.69–1.04)	0.74 (0.60–0.92)	0.63 (0.50–0.80)	0.44 (0.32–0.62)	<0.0001	0.85 (0.69–1.04)

* Model 1 was adjusted for age (years) and sex (men, women). ^†^ Model 2 was adjusted for residential area (rural, urban), educational level (<7 years, 7~12 years, >12 years), family T2D history (yes or no), hypertension diagnosis (yes or no), total energy intake (kcal/d), menopausal status (yes or no), hormone replacement therapy use (yes or no), and fasting glucose level (mg/dL) in addition to Model 1 covariates. Abbreviations: T2D, type 2 diabetes; HR, hazard ratio; CI, confidence interval.

**Table 3:** Association between healthy lifestyle score and incident T2D stratified by potential risk factors *.

		Healthy Lifestyle Score						
	No. of Cases/Person-Years	0~1	2	3	4	5	*p* for Trend	*p* for Interaction
Age, years								0.170
<49	467/47,618	ref	0.86 (0.65–1.13)	0.66 (0.50–0.89)	0.57 (0.40–0.81)	0.18 (0.07–0.44)	<0.0001	
≥49	756/50,783	ref	0.88 (0.63–1.23)	0.84 (0.60–1.16)	0.71 (0.50–1.00)	0.59 (0.38–0.91)	0.002	
Sex								0.941
Men	629/46,621	ref	0.82 (0.65–1.04)	0.76 (0.60–0.96)	0.64 (0.48–0.86)	0.52 (0.28–0.95)	0.001	
Women	594/51,781	ref	0.96 (0.51–1.81)	0.81 (0.44–1.48)	0.70 (0.38–1.29)	0.47 (0.24–0.92)	<0.0001	
Family T2D history								0.560
No	1046/88,131	ref	0.84 (0.67–1.05)	0.76 (0.61–0.95)	0.63 (0.49–0.81)	0.48 (0.33–0.68)	<0.0001	
Yes	177/10,271	ref	0.88 (0.52–1.51)	0.62 (0.36–1.05)	0.65 (0.37–1.16)	0.22 (0.06–0.78)	0.015	
Hypertension status								0.771
No	933/86,676	ref	0.84 (0.67–1.06)	0.73 (0.58–0.93)	0.64 (0.49–0.83)	0.48 (0.33–0.71)	<0.0001	
Yes	290/11,725	ref	0.89 (0.55–1.44)	0.81 (0.51–1.31)	0.64 (0.37–1.09)	0.40 (0.19–0.85)	0.005	
BMI, kg/m^2^								0.170
<25	473/58,148	ref	1.30 (0.67–2.52)	1.00 (0.52–1.92)	0.98 (0.51–1.90)	0.81 (0.40–1.65)	0.062	
≥25	750/40,253	ref	0.88 (0.69–1.11)	0.98 (0.76–1.24)	0.94 (0.69–1.27)	NA ^†^	0.939	
Anti-diabetic medication *								0.011
No	309/89,948	ref	0.69 (0.45–1.06)	0.78 (0.52–1.17)	0.59 (0.37–0.94)	0.48 (0.26–0.90)	0.025	
Yes	914/8453	ref	1.07 (0.84–1.37)	1.03 (0.80–1.32)	1.00 (0.77–1.31)	1.34 (0.88–2.04)	0.809	
Residential area								0.916
Rural (Ansung)	681/45,936	ref	0.84 (0.56–1.26)	0.78 (0.53–1.15)	0.66 (0.44–0.99)	0.50 (0.31–0.82)	0.001	
Urban (Ansan)	542/52,466	ref	0.85 (0.66–1.09)	0.67 (0.52–0.88)	0.60 (0.44–0.83)	0.34 (0.17–0.68)	<0.0001	

* Models were adjusted for age (years), residential area (rural, urban), educational level (<7 years, 7~12 years, >12 years), sex (men, women), family history of T2D (yes or no), hypertension diagnosis (yes or no), total energy intake (kcal/d), menopausal status (yes or no), hormone replacement therapy use (yes or no), and fasting glucose level (mg/dL). The stratifying variable in each subgroup analysis was not included as a covariate in the adjustment set. Abbreviations: T2D, type 2 diabetes; BMI, body mass index. ^†^ NA estimates for the maximum HLS category in the BMI 25 kg/m^2^ group, which occurred due to insufficient events in the stratum. * Anti-diabetic medication refers to medication use among participants who had newly developed diabetes during the follow-up period.

**Table 4:** Sensitivity analyses of the association between healthy lifestyle score and incident T2D risk.

		Healthy Lifestyle Score					
	No. of Cases/ Person-Years	0~1	2	3	4	5	*p* for Trend
Replacing hPDI with uPDI							
Model 1 *	1223/98,401	ref	0.69 (0.56–0.85)	0.58 (0.48–0.72)	0.38 (0.30–0.48)	0.27 (0.19–0.39)	<0.0001
Model 2 ^†^	1223/98,401	ref	0.84 (0.67–1.04)	0.77 (0.62–0.96)	0.56 (0.44–0.71)	0.43 (0.30–0.62)	<0.0001
2-year lag analysis							
Model 1 *	1047/98,077	ref	0.60 (0.49–0.75)	0.51 (0.42–0.63)	0.37 (0.29–0.46)	0.24 (0.17–0.35)	<0.0001
Model 2 ^†^	1047/98,077	ref	0.77 (0.62–0.96)	0.67 (0.54–0.83)	0.55 (0.43–0.70)	0.34 (0.23–0.49)	<0.0001
4-year lag analysis							
Model 1 *	907/97,540	ref	0.55 (0.44–0.70)	0.49 (0.39–0.61)	0.37 (0.29–0.47)	0.23 (0.16–0.34)	<0.0001
Model 2 ^†^	907/97,540	ref	0.67 (0.53–0.85)	0.61 (0.48–0.77)	0.52 (0.40–0.67)	0.30 (0.20–0.45)	<0.0001

* Model 1 was adjusted for age (years) and sex (men, women). ^†^ Model 2 was adjusted for residential area (rural, urban), educational level (<7 years, 7~12 years, >12 years), family T2D history (yes or no), hypertension diagnosis (yes or no), total energy intake (kcal/d), menopausal status (yes or no), hormone replacement therapy use (yes or no), and fasting glucose level (mg/dL) in addition to Model 1 covariates. Abbreviations: T2D, type 2 diabetes; hPDI, healthy plant-based diet index; uPDI, unhealthy plant-based diet index.

**Table 5:** HR (95% CI) for incident T2D by healthy lifestyle score, leaving out one lifestyle factor at a time *.

	HR (95% CI)	*p* for Trend
Smoking	0.65 (0.51–0.81)	<0.0001
Physical activity	0.51 (0.39–0.90)	<0.0001
Drinking	0.48 (0.36–0.65)	<0.0001
BMI	0.82 (0.65–1.04)	0.2145
Diet	0.42 (0.32–0.53)	<0.0001

* Adjusted for age (years), sex (men, women), residential area (rural, urban), educational level (<7 years, 7~12 years, >12 years), family T2D history (yes or no), hypertension diagnosis (yes or no), total energy intake (kcal/d), menopausal status (yes or no), hormone replacement therapy use (yes or no), and fasting glucose level (mg/dL). Abbreviations: T2D, type 2 diabetes; HR, hazard ratio; CI, confidence interval; BMI, body mass index.

## Data Availability

Data underlying the results of our study are not publicly available due to KoGES data policy. Data are available from the Division of Genetic Epidemiology and Health Index, NIH, Korea Disease Control and Prevention Agency (contact via So Ra Kwon at sora9360@korea.kr) for researchers who meet the criteria for access to confidential data.

## Supplementary Materials

The following supporting information can be downloaded at https://www.mdpi.com/article/10.3390/nu18020273/s1, Table S1: Baseline characteristics of participants by inclusion status.

## References

[1] Petersmann A., Müller-Wieland D., Müller U.A., Landgraf R., Nauck M., Freckmann G., Heinemann L., Schleicher E. (2019). Definition, classification and diagnosis of diabetes mellitus. Exp. Clin. Endocrinol. Diabetes.

[2] Magliano D.J., Boyko E.J. (2025). IDF Diabetes Atlas.

[3] (2010). Emerging Risk Factors Collaboration. Diabetes mellitus, fasting blood glucose concentration, and risk of vascular disease: A collaborative meta-analysis of 102 prospective studies. Lancet.

[4] Khan T.A., Field D., Chen V., Ahmad S., Mejia S.B., Kahleová H., Rahelić D., Salas-Salvadó J., Leiter L.A., Uusitupa M. (2023). Combination of multiple low-risk lifestyle behaviors and incident type 2 diabetes: A systematic review and dose–response meta-analysis of prospective cohort studies. Diabetes Care.

[5] Wu Y., He X., Zhou J., Wang Y., Yu L., Li X., Liu T., Luo J. (2022). Impact of healthy lifestyle on the risk of type 2 diabetes mellitus in southwest China: A prospective cohort study. J. Diabetes Investig..

[6] Zhang Y., Pan X.-F., Chen J., Xia L., Cao A., Zhang Y., Wang J., Li H., Yang K., Guo K. (2020). Combined lifestyle factors and risk of incident type 2 diabetes and prognosis among individuals with type 2 diabetes: A systematic review and meta-analysis of prospective cohort studies. Diabetologia.

[7] Bellou V., Belbasis L., Tzoulaki I., Evangelou E. (2018). Risk factors for type 2 diabetes mellitus: An exposure-wide umbrella review of meta-analyses. PLoS ONE.

[8] Hu F.B., Manson J.E., Stampfer M.J., Colditz G., Liu S., Solomon C.G., Willett W.C. (2001). Diet, lifestyle, and the risk of type 2 diabetes mellitus in women. N. Engl. J. Med..

[9] Lv J., Yu C., Guo Y., Bian Z., Yang L., Chen Y., Hu X., Hou W., Chen J., Chen Z. (2017). China Kadoorie Biobank Collaborative Group. Adherence to a healthy lifestyle and the risk of type 2 diabetes in Chinese adults. Int. J. Epidemiol..

[10] Han X., Wei Y., Hu H., Wang J., Li Z., Wang F., Long T., Yuan J., Yao P., Wei S. (2020). Genetic risk, a healthy lifestyle, and type 2 diabetes: The Dongfeng-Tongji cohort study. J. Clin. Endocrinol. Metab..

[11] Simmons R., Harding A.-H., Jakes R., Welch A., Wareham N., Griffin S. (2006). How much might achievement of diabetes prevention behaviour goals reduce the incidence of diabetes if implemented at the population level?. Diabetologia.

[12] Ford E.S., Bergmann M.M., Kröger J., Schienkiewitz A., Weikert C., Boeing H. (2009). Healthy living is the best revenge: Findings from the European Prospective Investigation into Cancer and Nutrition-Potsdam study. Arch. Intern. Med..

[13] Steinbrecher A., Morimoto Y., Heak S., Ollberding N.J., Geller K.S., Grandinetti A., Kolonel L.N., Maskarinec G. (2011). The preventable proportion of type 2 diabetes by ethnicity: The Multiethnic Cohort. Ann. Epidemiol..

[14] Djousse L., Driver J., Gaziano J., Buring J., Lee I. (2013). Association between modifiable lifestyle factors and residual lifetime risk of diabetes. Nutr. Metab. Cardiovasc. Dis..

[15] Lindström J., Peltonen M., Eriksson J., Ilanne-Parikka P., Aunola S., Keinänen-Kiukaanniemi S., Uusitupa M., Tuomilehto J., Finnish Diabetes Prevention Study (DPS) (2013). Improved lifestyle and decreased diabetes risk over 13 years: Long-term follow-up of the randomised Finnish Diabetes Prevention Study (DPS). Diabetologia.

[16] Tatsumi Y., Ohno Y., Morimoto A., Nishigaki Y., Mizuno S., Watanabe S. (2013). Lifestyle and the risk of diabetes mellitus in a Japanese population. J. Behav. Med..

[17] Long G., Johansson I., Rolandsson O., Wennberg P., Fhärm E., Weinehall L., Griffin S., Simmons R., Norberg M. (2015). Healthy behaviours and 10-year incidence of diabetes: A population cohort study. Prev. Med..

[18] Rajaobelina K., Dow C., Mancini F.R., Dartois L., Boutron-Ruault M.C., Balkau B., Bonnet F., Fagherazzi G. (2019). Population attributable fractions of the main type 2 diabetes mellitus risk factors in women: Findings from the French E3N cohort. J. Diabetes.

[19] Satija A., Bhupathiraju S.N., Rimm E.B., Spiegelman D., Chiuve S.E., Borgi L., Willett W.C., Manson J.E., Sun Q., Hu F.B. (2016). Plant-based dietary patterns and incidence of type 2 diabetes in US men and women: Results from three prospective cohort studies. PLoS Med..

[20] Thompson A.S., Candussi C.J., Tresserra-Rimbau A., Jennings A., Bondonno N.P., Hill C., Sowah S.A., Cassidy A., Kühn T. (2024). A healthful plant-based diet is associated with lower type 2 diabetes risk via improved metabolic state and organ function: A prospective cohort study. Diabetes Metab..

[21] Kim J., Giovannucci E. (2022). Healthful plant-based diet and incidence of type 2 diabetes in Asian population. Nutrients.

[22] Tan K.C.B. (2004). Appropriate body-mass index for Asian populations and its implications for policy and intervention strategies. Lancet.

[23] Lee H.S., Kim Y., Park T. (2018). New common and rare variants influencing metabolic syndrome and its individual components in a Korean population. Sci. Rep..

[24] Jacobs S., Harmon B.E., Boushey C.J., Morimoto Y., Wilkens L.R., Le Marchand L., Kröger J., Schulze M.B., Kolonel L.N., Maskarinec G. (2015). A priori-defined diet quality indexes and risk of type 2 diabetes: The Multiethnic Cohort. Diabetologia.

[25] Kim Y., Han B.G., KoGES Group (2017). Cohort profile: The Korean genome and epidemiology study (KoGES) consortium. Int. J. Epidemiol..

[26] Freisling H., Viallon V., Lennon H., Bagnardi V., Ricci C., Butterworth A.S., Sweeting M., Muller D., Romieu I., Bazelle P. (2020). Lifestyle factors and risk of multimorbidity of cancer and cardiometabolic diseases: A multinational cohort study. BMC Med..

[27] Reis J.P., Loria C.M., Sorlie P.D., Park Y., Hollenbeck A., Schatzkin A. (2011). Lifestyle factors and risk for new-onset diabetes: A population-based cohort study. Ann. Intern. Med..

[28] World Health Organization (2018). Global Status Report on Alcohol and Health 2018.

[29] Korean Society for the Study of Obesity (2018). Guideline for the Management of Obesity 2018.

[30] Ahn Y., Kwon E., Shim J.E., Park M.K., Joo Y., Kimm K., Park C., Kim D.H. (2007). Validation and reproducibility of food frequency questionnaire for Korean genome epidemiologic study. Eur. J. Clin. Nutr..

[31] Kim H., Lee K., Rebholz C.M., Kim J. (2020). Plant-based diets and incident metabolic syndrome: Results from a South Korean prospective cohort study. PLoS Med..

[32] American Diabetes Association (2024). Diagnosis and classification of diabetes: Standards of care in diabetes—2024. Diabetes Care.

[33] Han H., Cao Y., Feng C., Zheng Y., Dhana K., Zhu S., Shang C., Yuan C., Zong G. (2022). Association of a healthy lifestyle with all-cause and cause-specific mortality among individuals with type 2 diabetes: A prospective study in UK Biobank. Diabetes Care.

[34] Ma Y., Chen Y., Ge A., Long G., Yao M., Shi Y., He X. (2024). Healthy lifestyle associated with dynamic progression of type 2 diabetes: A multi-state analysis of a prospective cohort. J. Glob. Health.

[35] Li Y., Schoufour J., Wang D.D., Dhana K., Pan A., Liu X., Song M., Liu G., Shin H.J., Sun Q. (2020). Healthy lifestyle and life expectancy free of cancer, cardiovascular disease, and type 2 diabetes: Prospective cohort study. BMJ.

[36] Wu X., Tjahyo A.S., Volchanskaya V.S.B., Wong L.H., Lai X., Yong Y.N., Song M., Liu G., Shin H.J., Sun Q. (2025). A legume-enriched diet improves metabolic health in prediabetes mediated through gut microbiome: A randomized controlled trial. Nat. Commun..

[37] Taylor R. (2024). Understanding the cause of type 2 diabetes. Lancet Diabetes Endocrinol..

[38] James N.M., Stanford K.I. (2025). Obesity and Exercise: New insights and perspectives. Endocr. Rev..

[39] Jayedi A., Soltani S., Emadi A., Zargar M.S., Najafi A. (2024). Aerobic exercise and weight loss in adults: A systematic review and dose-response meta-analysis. JAMA Netw. Open.

[40] Fernández-Fígares Jiménez M.C. (2024). Plant foods, healthy plant-based diets, and type 2 diabetes: A review of the evidence. Nutr. Rev..

[41] Huh Y., Han K., Choi M.-J., Kim J.H., Kim S.M., Nam G.E. (2022). Association of smoking status with the risk of type 2 diabetes among young adults: A nationwide cohort study in South Korea. Nicotine Tob. Res..

[42] Yang B.C., Wu S.Y., Leung P.S. (2020). Alcohol ingestion induces pancreatic islet dysfunction and apoptosis via mediation of FGF21 resistance. Ann. Transl. Med..

[43] Hong S.-W., Linton J.A., Shim J.-Y., Kang H.-T. (2015). High-risk drinking is associated with a higher risk of diabetes mellitus in Korean men: Based on the 2010–2012 KNHANES. Alcohol.

[44] Yu H.-J., Ho M., Liu X., Yang J., Chau P.H., Fong D.Y.T. (2022). Association of weight status and the risks of diabetes in adults: A systematic review and meta-analysis of prospective cohort studies. Int. J. Obes..

[45] Luo J., Hodge A., Hendryx M., Byles J.E. (2021). BMI trajectory and subsequent risk of type 2 diabetes among middle-aged women. Nutr. Metab. Cardiovasc. Dis..

[46] Samuel V.T., Shulman G.I. (2016). The pathogenesis of insulin resistance: Integrating signaling pathways and substrate flux. J. Clin. Investig..

[47] Wang Y., Liu B., Han H., Hu Y., Zhu L., Rimm E.B., Hu F.B., Sun Q. (2023). Associations between plant-based dietary patterns and risks of type 2 diabetes, cardiovascular disease, cancer, and mortality: A systematic review and meta-analysis. Nutr. J..

