# Supplementary material for: The Association Between Composite Healthy Lifestyle Score and Type 2 Diabetes Risk in the Korean Population: The Korean Genome and Epidemiology Study
Source: Nutrients. 2026 Jan 14;18(2):273. doi: 10.3390/nu18020273 (PMC12845473; doi:10.3390/nu18020273)
Supplement: Supplementary file 1 [file nutrients-18-00273-s001.zip › nutrients-4029431-supplementary.pdf]

Table S1 Baseline characteristics of participants by inclusion status*

		Overall cohort	Included	P-value
**Participants**		10,030	7,185	
**Age, years**		52.3 (8.9)	51.6 (8.7)	<0.0001
**Sex**				0.2230
	Women	5,272 (52.56)	3,709 (51.62)	
**Smoking**				0.9904
	Never	5,808 (58.69)	4,223 (58.78)	
	Former	1,539 (15.55)	1,118 (15.56)	
	Current	2,549 (25.76)	1,844 (25.66)	
**Physical activity**				0.0830
	0	3,296 (32.86)	2,246 (31.26)	
	< 30 min/d	984 (9.81)	714 (9.94)	
	≥ 30 min/d	5,750 (57.33)	4,225 (58.80)	
**Alcohol consumption, g/d**				0.8968
	Men	25.3 (30.8)	25.5 (29.8)	
	Women	5.4 (11.5)	5.1 (9.8)	
**BMI, kg/m^2**		24.6 (3.1)	24.5 (3.1)	0.4728
**hPDI, score**		51.1 (6.5)	51.4 (6.3)	0.1731
**Fasting glucose, mg/dL**		92.4 (22.7)	88.3 (9.5)	<0.0001

* Data are expressed as n (%) or mean (SD). Abbreviations: BMI, body mass index; hPDI, healthy plant-based diet index
